# Effects of preoperative feeding with a whey protein plus carbohydrate drink on the acute phase response and insulin resistance. A randomized trial

**DOI:** 10.1186/1475-2891-10-66

**Published:** 2011-06-13

**Authors:** Francine Perrone, Antônio C da-Silva-Filho, Isa F Adôrno, Nadia T Anabuki, Fernando S Leal, Tariane Colombo, Benedito D da Silva, Diana B Dock-Nascimento, Aderson Damião, José E de Aguilar-Nascimento

**Affiliations:** 1 Department of Surgery, Julio Muller University Hospital, Federal University of Mato Grosso, Brazil; 2 Rodovia Helder Candia, Condominio Country 15 78048-150, Cuiabá, MT, Brazil

**Keywords:** preoperative fasting, insulin resistance, carbohydrates, whey protein, inflammatory response

## Abstract

**Background:**

Prolonged preoperative fasting increases insulin resistance and current evidence recommends carbohydrate (CHO) drinks 2 hours before surgery. Our hypothesis is that the addition of whey protein to a CHO-based drink not only reduces the inflammatory response but also diminish insulin resistance.

**Methods:**

Seventeen patients scheduled to cholecystectomy or inguinal herniorraphy were randomized and given 474 ml and 237 ml of water (CO group) or a drink containing CHO and milk whey protein (CHO-P group) respectively, 6 and 3 hours before operation. Blood samples were collected before surgery and 24 hours afterwards for biochemical assays. The endpoints of the study were the insulin resistance (IR), the prognostic inflammatory and nutritional index (PINI) and the C-reactive protein (CRP)/albumin ratio. A 5% level for significance was established.

**Results:**

There were no anesthetic or postoperative complications. The post-operative IR was lower in the CHO-P group when compared with the CO group (2.75 ± 0.72 vs 5.74 ± 1.16; p = 0.03). There was no difference between the two groups in relation to the PINI. The CHO-P group showed a decrease in the both CRP elevation and CRP/albumin ratio (p < 0.05). The proportion of patients who showed CRP/albumin ratio considered normal was significantly greater (p < 0.05) in the CHO-P group (87.5%) than in the CO group (33.3%).

**Conclusions:**

Shortening the pre-operative fasting using CHO and whey protein is safe and reduces insulin resistance and postoperative acute phase response in elective moderate operations.

**Trial registration:**

ClinicalTrail.gov NCT01354249

## Introduction

Perioperative care has been the subject of a number of studies in the last decade. The benefits of 6-8 hours of preoperative fasting in order to reduce the risk of pulmonary aspiration of gastric content [1] has been recently questioned by various studies [2-4]. Despite this, the "nothing by mouth after midnight" routine is still prescribed by many surgeons and anesthesiologists due to outdated concepts and paradigms [5]. In addition, conventional fasting is aggravated by the fact this it is usually prolonged. When added up, pre-operative fasting can be excessively long lasting form 10 up to 16 hours [6]. This may impair the recovery of patients since the organic response to surgical trauma is enhanced by the prolonged period of fasting.

Hyperglycaemia is a sensitive marker of the metabolic response to trauma, and it is due to a reduction of the peripheral action of insulin mediated by contra-regulating hormone action. These hormones stimulated glycogen and lipid catabolism thus stimulating hepatic neoglycogenesis as well as increasing peripheral resistance to insulin [7]. This response is related to the magnitude of the trauma as well as the duration of pre-operative fasting [7-9].

Trauma, as well as other causes of injury including fasting, induces a response typified by the release of inflammatory mediators. These mediators, including interleukins (IL) 1, 2, 6 and the tumor necrosis factor (TNF), appear to be related to the metabolic alterations found early after injury [8,9]. A significant increase in the IL-6 plasma concentration thirty minutes after surgery confirms that this cytokine is an early and sensitive marker of tissue lesion, and is related to the trauma magnitude and duration [10]. Besides IL-6, acute-phase proteins such as C-reactive protein (CRP), and negatives such as albumin and pre-albumin, may be useful as predictors of post-operative infection [8]. Within this context, the prognostic inflammatory and nutritional index (PINI) proposed by Ingenbleek and Carpentier has been used and validated to predict risks related to morbidity and mortality. The PINI uses a combination of two positive acute-phase proteins (CRP and α-1-acid glycoprotein) and two negatives (albumin and pre-albumin) [11]. Some studies have shown that formulas using acute-phase proteins such as the PCR/albumin ratio may predict risks for hospitalized patients [12,13].

To abbreviate preoperative fasting, beverages containing carbohydrates have been used and recommended [4,14-16]. Recently formulas containing either proteins or amino acids in addition to the carbohydrate-enriched drink have been proposed. These new formulas may improve post-operative muscle strength, reduce fatigue, anxiety and discomfort as well as lowering the endocrine-metabolic response to trauma [15]. The use of glutamine in the composition of these drinks improved the hepatic and mitochondrial metabolic response in two recent studies [16,17].

Whey protein contains a high level of essential amino acids especially branched-chain amino acids [18]. The branched-chain amino acids (leucine, isoleucine, and valine) are rapidly used by skeletal muscle during stress and highly stimulate protein synthesis [19,20]. In addition, they are precursors of endogenous synthesis of glutamine, the main energy source for enterocytes [19]. Whey protein has also been described as an excellent source of cysteine, a precursor of glutathione synthesis, which acts as an endogenous antioxidant [21]. Moreover, whey protein has a high degree of digestibility and rapid absorption in the small bowel [22]. No study so far has aimed to examine the possible benefits of whey protein in the composition of preoperative drinks. Therefore, the aim of this study was to investigate whether the shortening of pre-operative fasting using a solution containing carbohydrates and whey proteins may alter the organ ic response to trauma, focusing on insulin resistance and acute-phase response in elective moderate operations.

## Materials and methods

This was a randomized, double-blind, clinical study carried out at the Julio Muller University Hospital (Mato Grosso State, Brazil). The study was approved by the hospital Research Ethics Committee registered under number 635/CEP-HUJM/09 and is in accordance with the ethics principals set out in the Helsinki Declaration (2000), and meets Brazilian national legal specifications. The study was registered in ClinicalTrails.gov under the number NCT01354249. External monitoring of the study was carried out by the Research Ethics Committee of the Julio Muller Hospital.

Inclusion criteria includes adults (18-65 years-old), of both sexes, and candidates to elective moderate operations such as open cholecystectomy, laparoscopic cholecystectomy and unilateral repair of inguinal hernia). Exclusion criteria were acute cholecystitis, diabetes mellitus, chronic kidney failure, chronic liver disease ot serum bilirubin greater than 2 mg/dL, body mass index (BMI) above 35 Kg/m^2^, American Anesthesiologists Association (ASA) score above 2, gastro-esophageal reflux, gastroparesis or intestinal obstruction. Patients with any non-compliance with the study protocol, or who had associated operations, or presented significant intraoperative occurrences, or experienced prolonged operations (lasting more than 3 hours) were also excluded.

Patient randomization was carried out on admission to the hospital using random numbers issued by a computer program [23]. For the randomization the precepts of the CONSORT flow diagram were followed [24].

The patients were randomized into two groups: the carbohydrate-protein group (CHO-P) and the control group (CO). The patients were given a specific drink to their group on the evening prior to surgery and three hours before the operation. The CHO-P group received 474 ml (evening drink) or 237 ml (3 h prior to operation drink) of a solution containing 14% whey protein (100% lactoalbumin), 86% carbohydrates (45% maltodextrine and 55% sucrose) and 0% lipids (Resource^® ^Breeze - Nestlé, São Paulo, Brasil) and the CO group received the same volume of water. All the patients fasted for solids for 6 hours from the operation.

On the day of the surgery and on the first postoperative day blood samples were collected for glucose, insulin, triglycerides, albumin, pre-albumin, CRP, and α-1-acid glycoprotein (α-1-GA) assays. Preoperative samples were collected at the infirmary approximately one hour before the patient was sent to the surgical center.

The HOMA-IR (Homeostasis Model Assessment-Insulin Resistance) equation was used as proposed by Matthews et al [25] to assess insulin resistance according to the formula: HOMA-IR= insulin (μU/mL) × glycaemia (mg/dL)/405. To assess inflammatory activity the PINI (CRP (mg/L) × α-1-GA (mg/L)/albumin (g/L) × pre-albumin (mg/L) and the CRP/albumin ratio were used [11].

Nutritional status was assessed by BMI and subjective global assessment [26]. The patients received intraoperatively a single dose of 2 g of intravenous cefazolin and were hydrated with 1000-1500 ml of Ringer's lactate during immediate postoperative period. Dipyrone (500 mg IV every 6 hours) was prescribed for pain and anti-emetics were used only when necessary.

### Statistical Method

The sampling calculation was based on a previous study [27]. A quantity of 8 cases in each study branch was judged to be sufficient to ensure 80% power (beta error) and 5% significance (alpha error) expecting a difference in 50% in in the insulin resistance (Homa equation). All the continuous data were initially analysed for homogeneity by the Levene test and for normality by the Kolmogorov-Smirnov test. The Student *t *test or Mann-Whitney test was then applied accordingly. A cut-off for normality was for serum CRP (48 mg/L) and CRP/albumin ratio (11) based on the overall mean of the data plus 1 standard deviation. A significance level of 5% (p < 0.05) was established. The data were presented as a mean and standard deviation or as a median and variance. All the calculations were made on a computer using the *Statistical Package for the Social Sciences *(SPSS) for *Windows *11. 0.

## Results

**Figure 1:**
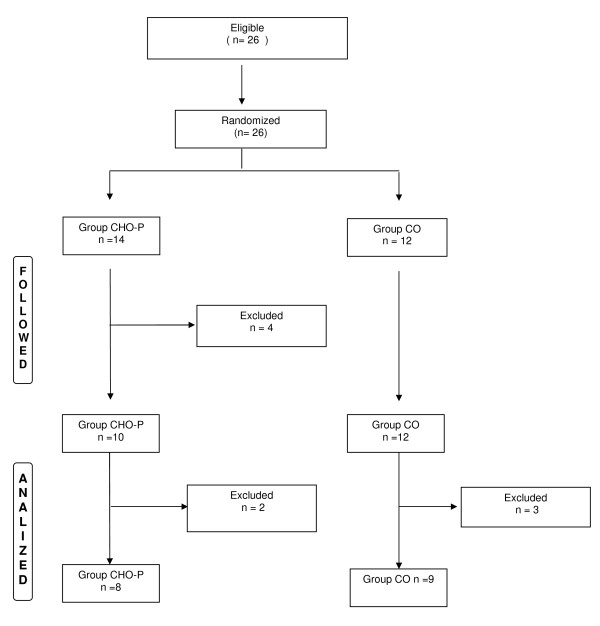
**Flowchart of randomization**.

Twenty six patients were eligible for the study and randomized to either CO group (n = 12) or CHO-P group (n = 14). Nine patients were excluded for different causes remaining eight patients in the CHO-P group and nine in the CO group. The flowchart of the study can be seen in Figure 1.

All the patients were classified as eutrophic. There were no deaths or postoperative complications in the two groups. The demographic and clinical characteristics of the patients can be seen in table 1.

**Table 1:** Demographics and clinical aspects of the patients in the two groups.

Variable	Group CO N (%)	Group CHO-P N (%)	p
Sex			
Male	3 (33.3)	3 (37.5)	1.00
Female	6 (66.7)	5 (62.5)	
Age (years) (mean ± SEM)	41.0 ± 4.0	35.0 ± 6.0	0.41
Type of operation			
Open cholecystectomy	6 (66.7)	4(50)	0.71
Video-cholecystectomy	2 (22.2%)	2 (25,0%)	
Inguinal hérnia repair	1 (11,1%)	2 (25,0%)	
Duration of the operation (min) (mean ± SEM)	99.0 ± 11.0	94.0 ± 8.0	0.52
Type of anaesthesia			
General	4 (44.4)	3 (37.5)	0.78
Epidural blockage	2 (22.2)	3 (37.5)	
General+ epidural blockage	3 (33.3)	2 (25)	
Duration of fasting (min) (mean ± SEM)	1014 ± 38	264 ± 9	0.001
Nutritional status			
Eutrophic	9 (100)	8 (100)	1.00
BMI* (kg/m^2^) (mean ± SEM)	30.7 ± 2.1	26.9 ± 1.5	0.19
ASA score^†^			
I	3 (33.3)	7 (87.5)	0.05
II	6 (66.7)	1(12.5)	
Length of hospital stay (median and range)	1 (1-2)	1(1-2)	0.40

* ^† ^Body mass index, ^† ^American Society of Aneshesiologists.

None of the patients presented anesthetic complications. Patients who received CHO-P beverage had no intolerance such as nausea or vomiting preoperatively.

### Glycaemia, insulin and insulin resistance

**Figure 2:**
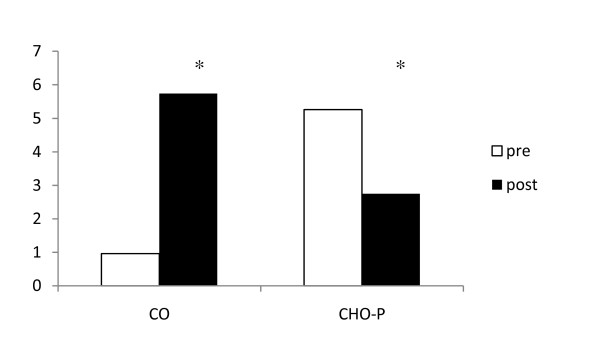
**Pre- and postoperative insulin resistance (HOMA-IR) in the two groups**. Data express the mean. * = p < 0.05 versus values of the preoperative period. CO = control group; CHO-P = whey protein group.

The CO group showed greater serum insulin (19.9 ± 3,99 versus 10.7 ± 2.9 μU/mL; p = 0.049) and insulin resistance (5.7 ± 1.1 versus 2.7 ± 0.7; p = 0.03) when compared to CO group postoperatively. The insulin resistance alterations before and after surgery were found to be significantly higher in the CO group as can be seen in Figure 2. There was also a trend to show higher glycaemia (115 ± 6 mg/dL) in this group as can be seen in table 2. Preoperatively the values of insulin resistance (5.3 ± 6.6 vs. 0.96 ± 0.27), glycaemia (103 ± 7 mg/dL vs 85 ± 5 mg/dL) and insulinemia (19.5 ± 5.1 μU/mL vs. 4.4 ± 1.2 μU/mL) were higher in the CHO-P group than in the CO group.

**Table 2:** Mean and standard error of the mean of postoperative serum glycaemia, insulin, and insulin resistance (HOMA-IR) in the two groups.

Variable	Group CO	Group CHO-P	P
HOMA-IR	5.74 ± 1.16	2.75 ± 0.72	0.03
Insulin	19.9 ± 3.9	10.7 ± 2.9	0.05
Glycaemia	115.0 ± 6.0	105.0 ± 8.0	0.09

CO = control group; CHO-P = whey protein group.

In the comparison between the alterations occurring in the two groups, between pre and post operative, significant differences were found in the three parameters. Thus, there was a significant increase (p < 0.05) in glycaemia, insulinemia and insulin resistance increase in the CO group and a reduction in the CHO-P group which can be clearly seen in table 3.

**Table 3:** Mean and standard error of the mean of the difference between preoperative and postoperative values of serum glycaemia, insulin, and insulin resistance (HOMA-IR) in the two groups.

Variable	Grupo CO	Grupo CHO-P	p
HOMA-IR	4.8 ± 1.1	-2.5 ± 1.5	p = 0.001
Insulin	15.5 ± 3.8	-8.8 ± 4.6	p < 0.001
Glycaemia	30.0 ± 7.3	1.6 ± 12.1	p = 0.036

CO = control group; CHO-P = whey protein group.

**Figure 3:**
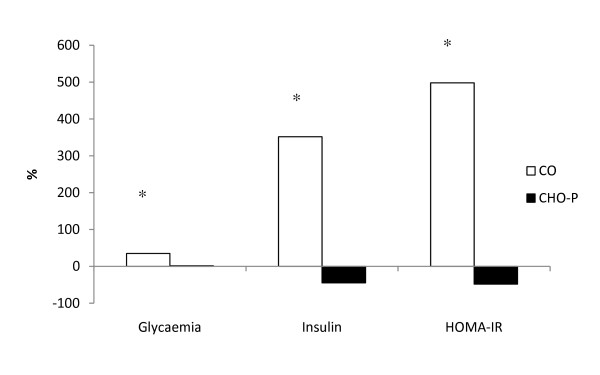
**Percentage changes between pre- and postoperative values of serum glycaemia, insulin, and insulin resistance (HOMA-IR) in the two groups**. * p < 0.05 *versus *group CHO-P. CO = control group; CHO-P = whey protein group.

Figure 3 mirrors the percentage variance that occurred with glycaemia, insulinemia and in the HOMA-IR before and after surgery. A significant difference was found between the two groups.

### Inflammatory response

The CRP values in the pre and post-operative periods for the two groups can be seen in table 4. Serum CRP increased significantly (p < 0.01) in the two groups. However, postoperative serum CRP was significantly higher in CO group. There were no cases in the CHO-P group of values above the cut-off point as shown in table 5 (p = 0.03).

**Table 4:** Mean and standard error of the mean of various inflammatory markers in the two groups.

Variable	Period and groups					
	**Preoperative**			**Postoperative**		

	**CO**	**CHO-P**	**p**	**CO**	**CHO-P**	**p**
	
Albumin (g/dL)	4,15 ± 0,13	4,26 ± 0,09	0,53	3,82 ± 0,12	4,02 ± 0,14	0,29
Prealbumin (mg/dL)	0,25 ± 0,02	0,22 ± 0,01	0,33	0,26 ± 0,05	0,21 ± 0,02	0,43
CRP (mg/L)	7,4 ± 1,27	6,25 ± 0,90	0,46	40,12 ± 10,04	27,37 ± 4,76	0,29
α-1-GA (mg/dL)	75,87 ± 3,94	98,1 ± 8,9	0,03	90,5 ± 3,9	105,8 ± 6,5	0,57
PINI	0,63 ± 0,16	0,76 ± 0,22	0,64	5,98 ± 2,05	3,93 ± 1,01	0,40
CRP/albumin ratio	1,81 ± 0,32	1,46 ± 0,20	0,38	10,99 ± 2,89	6,97 ± 1,35	0,25

CO = control group; CHO-P = whey protein group; CRP = C reactive protein; α-1-GA = α-1-acid glycoprotein; PINI: prognostic inflammatory and nutritional index

**Table 5:** Postoperative serum C-reactive protein (CRP) below or above the cutoff point (48 mg/L) in the two groups.

CRP	Group CO N(%)	Group CHO-P N(%)
Above	5(55.5)*	0 (0)
Below	4 (44.5)	8 (100)
Total	9	8

CO = control group; CHO-P = whey protein group.

*p = 0,03

Preoperatively serum α-1-acid glycoprotein was greater (p = 0.03) in the CHO-P group (98.1 ± 8.9 mg/dL) than in the CO group (75.8 ± 3.9 mg/dL). However, there was no significant difference between the groups (p = 0.57) at the postoperative period, as can be seen in table 4. Serum α-1-acid glycoprotein significantly increased only the CO group (p < 0.01).

There were no differences in the albumin values between the two groups in both the pre (p = 0.53) and post-operative assays (p = 0.29). However CO group showed a significant decrease from pre- to postoperative values (p < 0.01).

There was no difference in serum pre-albumin levels in the two groups. The CRP/albumin ratio increased after the operation in both groups. However the number of cases with CRP/albumin ratio above the cutoff was greater in CO groups when compared to CHO-P group (p = 0.04) as shown in table 6.

**Table 6:** Postoperative serum C-reactive protein (CRP)/albumin ratio below or above the cutoff point (11) in the two groups

CRP/albumin ratio	Group CO N(%)	Group CHO-P N(%)
Above	6 (66.7)*	1 (12.5)
Below	3 (33.3)	7 (87.5)
Total	9	8

CO = control group; CHO-P = whey protein group.

.*, p = 0,04

## Discussion

The overall results showed that the postoperative peripheral insulin resistance was reduced with the abbreviation of fasting with a beverage containing carbohydrates plus whey protein. Insulin resistance is a transitory phenomenon restricted to the first few days after surgery and lasting up to three weeks in cases of elective abdominal operations with no complications [10]. This metabolic state is very similar to type II diabetes whereby glucose capture by cells is reduced due to the incapacity of the GLUT-4 transporter to carry out this function and, consequently, the production of glycogen is reduced [10]. Simultaneously, there is an increase in the endogenous glucose that may become a serious risk factor for increase morbidity [28]. Thus the findings of increased insulin resistance after the operation in the group submitted to prolonged fasting was expected. This result contrasts with the opposite evolution seen in the CHO-P groups and was similar in other studies [27,29]. At the preoperative phase the CHO-P group presented an increased HOMA-IR in comparison to CO group. However this was expected because blood samples were collected one hour before the patients had entered the operating room and after they had ingested the preconditioning beverage.

Proteins and amino acids may reduce the insulin response in some clinical conditions such as obesity and type II diabetes [30,31]. Whey protein is a rich source of essential amino acids which are the most important regulators of this response [32]. Indeed, the addition of whey protein to a carbohydrate drink improved the insulin response compared with the ingestion of carbohydrates only [33]. However the findings of this study should be seen with the necessary caution because there was not a group with only whey protein and thus the results seen here may theoretically reflected only the CHO effect.

There was a delay from the programmed preoperative fasting time in both the two groups. Indeed CHO-P patients were operated on after approximately 4 h of fasting and CO group after 17 h. This frequent delay was reported earlier by our group [6,27]. It should be in the mind of both the surgeon and the anesthesiologist that this extended fasting is most prone to occur and therefore should be audit in every institution due to the harm that it may cause to the patients.

The type of CHO contained in the beverage tested was a mixture of maltodextrine and sucrose. Maltodextrin is a sweat, easily digested carbohydrate made from corn starch. It contains small chains of several dextrose molecules held together by very weak hydrogen bonds. This makes its absorption slower than sucrose and thus elicits maltodextrin as an excellent CHO for preoperative drinks. The solution containing both maltodextrine and sucrose may enhance the activation of more transport mechanisms in the intestinal lumen and for this reason may facilitate faster energy uptake and hydration [34].

CRP is a positive acute-phase protein and its levels well correlate with the inflammation intensity and may predict postoperative complications [1,8]. In opposition a decrease of negative acute-phase protein such as albumin and prealbumin after trauma is expected due to the inhibition of their synthesis by the pro-inflammatory cytokines [7]. The findings have shown that the inflammatory response to trauma was less intense in the intervention group. Significantly changes of serum albumin levels and serum α-1-GA were only found in the CO group. Besides this, the proportion of cases with abnormal CRP levels was greater in the control group. These findings are relevant and suggest that a lower inflammatory response was presented in the group fed with carbohydrates plus whey protein 3-4 h prior to surgery.

The genetic expression of α-1-GA is controlled by a combination of the main inflammatory mediators such as glycocorticoids and a network of cytokines [35]. At the preoperative sample we found an increased serum α-1-GA in the CHO-P group when compared to CO group. This result seen in whey protein fed patients was probably due to the early supply of lactoalbumin. Genes that codify the whey protein are similar to the genes that codify the α-1-GA [36]. Thus, the supply of whey protein, especially lactoalbumin may have contributed to the increase in the α-1-GA concentration level. However, only CO group showed a significant increase in serum α-1-GA between the pre- and postoperative sam[ples suggesting a greater inflammatory response. However, the PINI calculation that uses the values of serum α-1-GA was probably biased.

One possible criticism for this study is the small number of cases. However, we performed sample calculation aiming for a power analysis above 80%. If a difference in treatment can be seeing with small sample (but with sufficient power analysis) adding subjects may only increase duration and costs of the study. We believe that samples should be large enough to detect possible differences, reasonable enough to be feasible, and small enough to detect efficient therapies. Another possible criticism is that ASA score was higher in CO group. However we excluded patients with important chronic diseases and only elicited in the study patients with ASA score I and II. We do not believe that this finding could compromise the results.

In summary the shortening of preoperative fasting to 3 hours using a drink containing carbohydrates and whey protein not only have minimized the postoperative insulin resistance but also reduced the magnitude of the acute phase inflammatory response. Thus we concluded that the abbreviation of preoperative fasting with carbohydrates plus whey protein is safe and diminishes the organic response after moderate operations in general surgery.

## Conflict of interests

The authors declare that they have no competing interests.

## Authors' contributions

FP - data collection; manuscript drafting and organization;

ACSF, IFA, NTA, FSL, TC, and BDO - sequence alignment, laboratory data and acquisition of the data. DBDN, AD, and JEAN - study design and concept; data analysis, manuscript drafting and organization. All authors have read and approved the final version of the manuscript.

## Funding

This study was funded by the *Fundação de Amparo à Pesquisa de Mato Grosso *and the *Coordenação de Aperfeiçoamento de Pessoal de Nível Superior*

## Acnowledgements

This study was funded by the FAPEMAT (*Fundação de Amparo à Pesquisa de Mato Grosso*) and by the CAPES (*Coordenação de Aperfeiçoamento de Pessoal de Nível Superior*).
